# Voice outcome in medialisation thyroplasty with and without arytenoid adduction: a prospective comparison using intraoperative voice measurements

**DOI:** 10.1007/s00405-024-08494-3

**Published:** 2024-02-17

**Authors:** S. D. Mes, M. A. van der Jagt, J. C. Jansen, A. P. M. Langeveld, E. V. Sjögren, B. J. Heijnen

**Affiliations:** 1 Department of Otolaryngology and Head- and Neck Surgery, Leiden University Medical Center, Albinusdreef 2, Postbus 9600, Leiden, RC The Netherlands; 2 Department of Otolaryngology, Alrijne Hospital, Leiden, The Netherlands

**Keywords:** Vocal fold paralysis, Medialisation thyroplasty, Arytenoid adduction, Voice outcome, VHI

## Abstract

**Purpose:**

Arytenoid adduction as an addition to medialisation thyroplasty is highly advocated by some surgeons in selected cases but deemed less necessary by others in patients with unilateral vocal fold paralysis. This study aims to evaluate the additional benefits on voice outcome of arytenoid adduction in patients with unilateral vocal fold paralysis undergoing medialisation thyroplasty using intra-operative voice measurements.

**Design/methods:**

A prospective study was conducted. Voice audio recordings were obtained at 4 moments; 1. direct prior to the start of surgery, 2. during surgery after medialisation thyroplasty, 3. during surgery after medialisation and arytenoid adduction, 3 months postoperative. At these same timepoints patients rated their own voice on a numeric rating scale between 0 and 10. The blinded recordings were rated by consensus in a team of experienced listeners, using the Grade of the GRBAS scale. Furthermore, the Voice Handicap Index was administered before and at 3 months after surgery.

**Results:**

Ten patients who underwent medialisation and arytenoid adduction at our tertiary referral hospital between 2021 and 2022, were included. One patient was excluded after surgery. The intraoperative measurements showed a Grade score of 1.4 preoperatively, improving to 1.2 after medialisation, 1.2 after medialisation and arytenoid adduction, and further improving to 0.4 at 3 months postoperative, which was a not statistically significant improvement (*p = *0.2). The intraoperative subjective numeric rating scale showed a statistically significant improvement from 3.9 preoperatively, to 6.1 after medialisation, 7.1 after medialisation and arytenoid adduction and a 7.6 at 3 months postoperative (*p = *0.001). The Voice Handicap Index total score showed a statistically significant improvement from 71 points before surgery to 13 at 3 months after surgery (*p = *0.008).

**Conclusions:**

Our study using intraoperative voice measurements indicate that the addition of arytenoid adduction to medialisation thyroplasty is a benefit in selected patients although more studies are needed due to the many limitations inherent to this field of investigation.

## Introduction

Unilateral vocal fold paralysis (UVFP) can have significant impact on functional health status and quality of life, due to its wide range of symptoms. The position of the paralyzed vocal fold determines the degree of glottal insufficiency, a wide glottal gap often leads to severe dysphonia. Several treatment modalities for UVFP were introduced in the mid-1970s. Nowadays laryngeal framework surgery is a standard treatment for UVFP and provides a durable solution to glottic insufficiency caused by injury to the recurrent laryngeal nerve [1]. Various modifications to the original Isshiki type I medialisation thyroplasty (MT) procedure have been described, including the arytenoid adduction (AA). This is a rotation of the arytenoid joint to move the vocal process medially, posteriorly and inferiorly, and thus also medializes the vocal fold [2]. Arytenoid adduction mimics the physiologic action of the lateral cricoarytenoid muscle of the larynx during phonation, but the disadvantage of AA is its inability to bulk up the anterior membranous vocal fold. Therefore, AA procedure is often performed in combination with MT [3, 4].

In general, arytenoid adduction is recommended for UVFP cases with a large posterior glottic gap or level difference between the vocal folds [5–8]. The decision to include arytenoid adduction as an additional procedure to medialisation thyroplasty depends on the surgeon’s experience, preference, and results from stroboscopic imaging and per-operative voice quality. However, even in a selected group of patients, the exact added value of arytenoid adduction remains unclear, as patients could possibly attain sufficient glottal closure with medialisation thyroplasty only [9].

Several studies have compared the effect of MT and the combination of MT + AA on voice quality. However, the results of these studies have been inconsistent, with some reporting greater improvements in one or more voice parameters following MT + AA compared to MT alone [7, 8, 10], while others have found no additional benefit from the addition of AA [9, 11–13]. Since these studies are evaluating both treatments in a retrospective manner, their results could be influenced by allocation bias. A second reason why comparing MT vs MT + AA is challenging is that not every UVFP has the same characteristics [14]. A paralyzed vocal fold can adapt a median, paramedian or lateral rest position [15]. As study groups are generally small, case mix may, therefore, be an explanation for the conflicting results as to the value of the additional AA.

Several authors have suggested that a study with intraoperative measurements of the results of MT and AA could reveal what the additional value of AA would be [7, 9, 16]. Therefore, in this study, voice outcome assessment was performed intraoperatively after medialisation only and after the addition of an arytenoid adduction to evaluate their individual contribution to voice outcome.

## Materials and methods

### Patients

This study was conducted prospectively in 10 patients who underwent MT + AA at our tertiary referral hospital between 2021 and 2022. All patients were diagnosed with UVFP by an experienced laryngologist based on clinical evaluation including videolaryngostroboscopy. Patients that were anticipated to need an arytenoid adduction due to the size and/or shape of the phonatory gap were asked to participate in the study. Data on patient etiology, medical history, demographics, complications and intraoperative voice recordings were collected. Comorbidity scores were rated using the Adult–Comorbidity–Evaluation-27 (ACE-27)[17]. Patients with a previous laryngeal framework surgery were excluded. Institutional review board approval for the publication of this data was obtained from the Leiden University Medical Centre Ethics Committee (G21.030).

### Voice data collection

Voice audio recordings were obtained at four timepoints; 1. Direct prior to the start of surgery, 2. during surgery after MT, 3. after surgery when MT + AA was completed, 4. 3 months postoperative. Three voice tasks that were recorded intraoperatively; 1. counting from 1 to 10, 2. naming the days of the week, 3. 20 s of spontaneous speech. For these intraoperative audio recordings, we used a portable field recorder (ZOOM, F1L, 96 kHz sample rate) with fixed distance cardioid headset (AKG, 644MD, 20–20.000 Hz). Voice samples were blinded for patient identity and timepoint to prevent observer bias. The recordings were rated using the overall grade (G) from the GRBAS rating scale, by consensus in a team of experienced (> 15 years) listeners (B.J.H. & V.A.H.vd K.) [18].

Complementary to these measurements, the patients were asked to give an overall grade for their voice on a numeric rating scale between (NRS) 1 and 10 (1 very poor, 10 outstanding). Although this is not a validated assessment tool, we chose to include it because it is a very familiar grading system for the Dutch population as it is the main system used in secondary and higher education. Scores range from 1 (very poor) to 10 (outstanding). A grade of 6 and above is considered a pass, 5 and below a fail. Grades nine and ten are seldom awarded. In general, an average grade of an 8 is considered a very good result. This rating system has also been described in one of our previous publications [16].

Furthermore, a standardized multidimensional voice data collection protocol was followed before treatment and 3 months after treatment, according to our standard clinical practice. At these timepoints, patients filled in a self-administrated questionnaire and speech samples were recorded. Patient self-assessment was performed using the Dutch validated version of the Voice Handicap Index questionnaire (VHI-30). A change of 10 points in an individual patient’s score was considered clinically relevant[19]. In addition, for these measurements the perceptual voice quality was evaluated by 2 experienced (> 15 years) listeners (B.J.H. & V.A.H.vd K.) using the overall grade (G) from the GRBAS rating scale. Recordings were made at a sampling frequency of 44.1 kHz with a dual range microphone, in a noise-free environment. Microphone to mouth distance is 10 cm. Speech material for perceptual analysis consists of a phonetically balanced Dutch text “80 dappere fietsers” [80 brave cyclists]. For this study, voice recordings were analysed using Audacity software (Boston, MA, 2016).

### Surgical technique

As described in our previous publication the surgical procedure at our institution is carried out under local anaesthesia using the standard technique described by Isshiki [20]. A window is drilled in the thyroid cartilage, using standardized window measurements and the inner perichondrium is released and usually incised posterior-caudally [21]. The implant is positioned and fixated after endoscopic evaluation of the window position. Implant material used in this study was Gore-Tex (GORE-TEX^®^ Soft Tissue Patch, Gore Medical, Flagstaff, Arizona). According to standard practice at our institution the likely need for AA is estimated preoperatively, but the definitive decision is made intraoperatively based on the voice results following trial MT. For AA, the thyroid cartilage is rotated to the contralateral side and the constrictor muscle is incised to expose the posterior margin. The cricothyroid joint is not routinely dislocated, neither the superior horn of the thyroid is transsected. The mucosa of the pyriform recess is elevated from the inner perichondrium through blunt dissection. After identification of the posterior cricoarytenoid muscle, the cricoarytenoid joint and muscular process of the arytenoid are identified. A suture (4.0 GORE-TEX^®^) is placed through the muscular process and guided ventrally along the inner perichondrium of the thyroid cartilage, while the joint stays intact. The decision as to the degree of suture traction and the degree of medialisation is based on three aspects of voice outcome which are evaluated intraoperatively: the perceptive voice outcome as judged by the surgeon and the patient, the ease of phonation as judged by the patient and the endoscopic assessment (videolaryngostroboscopy) by the surgeon during phonation. All patients had four days of voice rest postoperatively. Subsequently, they received voice therapy by an experienced speech-language therapist in the first week after surgery. Voice therapy included resonance exercises and vocal hygiene advices[16].

### Statistical analysis

All data were analysed using SPSS (IBM SPSS Statistics for Windows, Version 25.0, IBM Corp, Armonk, NY, USA). Statistical analysis of the Voice Handicap Index improvement was performed using the Related Samples Wilcoxon Signed Rank Test. Statistical analysis of Grade of GRBAS scale and NRS on voice during surgery was performed using the Friedman’s ANOVA. For the statistical tests, a *p* value < 0.05 was considered significant. For the NRS grade a post hoc analysis using the Wilcoxon signed rank test with a Bonferroni adjustment was applied to prevent a type 1 error in multiple comparisons. The adjusted *p* value indicating significant difference was *p* < 0.0125.

## Results

### Patients

In total, 10 patients were treated with MT + AA during the study period and underwent perioperative data collection. One patient was excluded after surgery after further EMG investigation showed that this patient had a contralateral vocal fold paresis. The etiology of the unilateral vocal fold paralysis was iatrogenic (*n* = 3), neoplasia (*n* = 3) or idiopathic (*n* = 3). The left vocal fold was affected in 4 patients and 5 patients had a right vocal fold paralysis. Mean age was 69 years (ranging from 52 to 91 years). This analysis included 3 male patients and 6 female patients. Mean ACE score was 1.8, therefore, varying from no to mild comorbidity. One patient had received injection augmentation medialisation with hyaluronic acid 16 months before laryngeal framework surgery. All attempted MT + AA surgeries were completed as planned, without complications.

### Perioperative assessment

Table 1 shows the individual grade scores on the GRBAS scale. The mean preoperative grade score was 1.4. During surgery, the grade score improved to 1.2 directly after medialisation and remained the same after arytenoid adduction. Three months postoperatively the grade had improved to a mean grade score to 0.4 (Table 1). The improvement of grade score over time was not statistically significant (*p = *0.2).

**Table 1:** Grade score of GRBAS scale

Grade score of GRBAS scale					
Patient	Preoperative	After medialisation	After medialisation + rotation	3 month postoperative	*p* value*
1	3	2	2	0	
2	1	2	1	1	
3	0	0	2	0	
4	2	1	1	1	
5	1	1	2	2	
6	0	1	1	0	
7	1	2	2	0	
8	3	2	0	0	
9	2	0	0	0	
Mean	1.4	1.2	1.2	0.4	0.2

^*^p value < 0.05 was considered significant

Table 2 shows the result of the overall rating by patients of their voice during surgery on a numeric rating scale from 0 to 10. This shows a mean preoperative score of 3.9. After MT the score was 6.1 and after MT + AA, it was 7.1. Three months postoperatively the score had further improved to 7.6 (Table 2). The Friedman’s ANOVA revealed that the improvement over time was statistically significant (*p = *0.001).

**Table 2:** Numeric rating scale subjective grades 0–10

Numeric rating scale subjective grade					
Patient	Preoperative	After medialisation	After medialisation + rotation	3 month postoperative	*p* value*
1	4.0	5.0	6.5	8.0	
2	6.0	8.0	8.0	8.0	
3	4.0	7.5	7.0	8.0	
4	2.0	5.0	6.0	8.0	
5	2.0	5.0	7.5	4.0	
6	7.0	5.5	6.5	8.0	
7	4.5	7.5	8.5	8.0	
8	4.0	6.0	7.0	8.0	
9	2.0	5.0	7.0	8.0	
Mean	3.9	6.1	7.1	7.6	0.001

^*^p value < 0.05 was considered significant

Post hoc analysis using the Wilcoxon signed rank test with a Bonferroni adjustment revealed that NRS grade did significantly improve from pre-operative to after MT + AA (*p = *0.011), and from pre-operative to 3 months postoperative (*p = *0.007), but not from preoperative to after MT alone (*p = *0.014), and not from MT to MT + AA (*p = *0.016). When using the Bonferroni adjustment for multiple comparisons a *p* value < 0.0125 was considered significant.

In the grade assessment, it is noteworthy that two patients (patient 3 and patient 6) have a preoperative grade score of 0. The reason for operating on patient 3 was not based on the perceptive deterioration of her voice, but rather on the fact that producing voice required a significant amount of effort. She had limited endurance and vocalizing was very fatiguing for her. Even though this improvement is not evident in the grade score, it is apparent in both the VHI score (improvement from 73 to 6) and the NRS scores (Table 2). Patient 6 also suffered primarily from vocal fatigue. At first evaluation her dysphonia was severe (grade 3) but after two hyaluronic acid injections (the latest being 14 months before MT + AA) her perceptive voice evaluation remained improved. However, limited endurance and vocal fatiguing was the reason she elected to undergo MT + AA surgery. In addition, this patient’s improvement is not evident in the grade score, but does show in the VHI score (improvement from 38 to 0) and the NRS scores (Table 2).

One patient (nr 5) exhibited a decrease in her score 3 months following the surgery as compared to their scores intraoperatively. This particular patient of 91 years had a decline in her vocal capabilities 6 week post-surgery which was related to a shift in position of the Gore-Tex although there was still sufficient closure on phonation. The patient elected to accept the current status and declined revision surgery.

### Pre- and postoperative assessment

#### Voice handicap score

Preoperatively, the mean VHI score was 71. The mean VHI score at 3 months after surgery was 13. All patients showed clinically relevant improvement (improvement of 10 points or more [19]), with a mean improvement of 58 points (ranging from 38 to 90 points). The improvement of VHI score postoperative was statistically significant (*p = *0.008).

## Discussion

Medialisation thyroplasty is an established surgical treatment for unilateral vocal fold paralysis. The addition of arytenoid adduction in selected cases is advocated by various surgeons to further improve outcome. However, it has been difficult to firmly establish added value of AA in scientific studies. This study describes a novel approach to this dilemma, for which we collected intraoperative voice data before and after AA as an alternative attempt to evaluate the added value of this procedure in combination with MT.

In the intraoperative self-ratings we found a mean improvement of 2.1 points on a 10 point scale after MT and then an additional 1.1 points of improvement after adding the AA. This shows that on average patients experienced subjective benefit of this additional procedure although the data showed considerable variability per patient. This variability is likely due to small sample size but surgical factors such as oedema on the vocal folds along with patient related factors such as fatigue, pulmonary status and stress together with the complexity of the task could also contribute to the variation. In addition, AA may not provide the same amount of benefit in every case.

The other parameter used in this study, the intraoperative Grade of the GRBAS score, showed no additional improvement after the addition of the AA. This does not necessarily contradict the subjective improvement experienced by patients as this could be mostly related to vocal effort as opposed to the perceptive quality of the voice. Interestingly, the assessment of the Grade of dysphonia at 3 months showed a further decrease of the intraoperative values supporting the earlier observation that the intraoperative environment is not the ideal assessment situation and that further improvements are likely after proper wound healing and speech-language therapy. The overall decrease of the Grade score from 1.4 before surgery to 0.4 at 3 month post-surgery (Δ 1.00 points; *p = *0.071), although not significant, is in line with earlier studies that have shown improvements in overall Grade score after MT + AA ranging from 1.0 to 2.5 points [8, 22–24]. Finally, the VHI total scores decreased significantly after surgery from a preoperative mean of 71 to 13 at 3 months (Δ 58; *p = *0.008) which can be considered as normal [22]. Various studies have used the VHI-30 score for voice outcome analysis in MT + AA cohorts reporting mean improvements ranging from 50.3 to 78.8 points [5, 23, 24].We, therefore, conclude that our data show a relevant voice improvement after MT + AA that is comparable with literature. However, in our case intra-operative measurements were not finite and continued to improve post-operatively in most cases.

The question arises as to whether the grade score we used, is the preferred indicator of voice outcome to measure this improvement, since it seems to have a floor- and ceiling effect and is a relatively coarse scale of only 4 outcomes. Given that this study represents an initial exploration involving intraoperative measurements, it was deemed an appropriate starting point. However, in further research, it would be beneficial to include additional voice outcome indicators, taking into consideration the limited tolerance of the patient during the surgery.

To the best of our knowledge, intraoperative voice recordings to compare the voice improvement after MT versus MT + AA in the same patient have not been reported before. However, intraoperative measurements for medialisation thyroplasty alone have been published. Two studies only performed intraoperative measurements and did not compare them to post-operative follow-up data. Ho et al. compared the effect of a VOIS^®^ implant to that of a titanium vocal fold medialisation (TVFM) implant in the same patient. The VOIS^®^ implant was implanted first and then replaced with a TVFM implant with intraoperative voice measurements being carried out for both implants. They used the R and B score of the GRBAS scale, MPT(maximum phonation time) and videolaryngostroboscopy as outcome measurements and found comparable improvement for the two implants[25]. Matar and Almohizea used intraoperative peak direct subglottic pressure recordings during MT with Montgomery implants and both authors found this an easy, feasible and useful tool to optimize their implant size during surgery [26, 27].

Two studies compared their intra-operative measurements to post-operative follow-up. Guzman et al. used a mobile voice laboratory to establish a voice spectrogram and assess fundamental frequency during MT with Gore-Tex^®^ implantation. They concluded that there were no significant differences in scores intraoperative directly after placing the implant, compared to 6 weeks after surgery, suggesting the reliability of the intraoperative improvement in their measurements [25]. Sanuki et al. used a multidimensional set of intraoperative voice measurements, including the GRBAS scale, perceptual evaluation and acoustic analyses for 18 adductor spasmodic dysphonia patients in which the effect of type 2 thyroplasty with titanium bridges was evaluated. They concluded that voice evaluations performed in the operating room can be considered equally valid when compared to those obtained in a soundproof room in the outpatient clinic obtained at 13 and 52 weeks [26].

From these limited studies using intraoperative voice measurements, no hard conclusions on their reliability to predict long-term outcome can be drawn, but the last study suggest that intraoperative measurements can be useful to evaluate the effect of thyroplasty surgery.

### Limitations

The first limitation of this study is the small sample size. Although intrinsic to this type of surgical procedure it will have an impact on the statistical power. Furthermore, the objective of this study was to prevent the potential selection bias which may arise when comparing MT to MT + AA. Although the definitive decision to perform AA was taken intraoperatively, when MT alone did not provide sufficient voice improvement. All patients included in this study were selected and counselled for possible MT + AA based on their preoperative stroboscopy indicating the possible need for this procedure. Therefore, this study does not prove that all patients benefit from an AA but indicates that it can be of added value in selected cases. In addition, the decision was made to keep the voice samples short to minimize burden on the patients during the surgery. This may result in potentially less assessable voice samples especially as in our experience this short evaluations were already quite taxing on the patients. In addition, we found considerable variability between patients where we suspect that supine position and possible discomfort along with stress, presence of mucus, the effect of sedative medication and the effect of (micro)edema due to manipulation of the arytenoid are all possible influences on the voice outcome which could make intraoperative evaluations less reliable. During the course of this study it also became clear that the surgeon is being challenged to listen “beyond” these possible deteriorating conditions to evaluate the change of voice outcome, and that, therefore, experience-based judgement plays a large role in this type of surgery.

## Conclusion

In UVFP patients with a large posterior glottic gap or a level difference of the vocal folds, an AA in combination with MT is often recommended for optimal voice improvement. Our study using intraoperative voice measurements indicate that the addition of AA to MT is a benefit in selected patients although more studies are needed due to the many limitations inherent to this field of investigation.

## Data Availability

Not applicable.
